# Crohn’s Disease Patients in Remission Display an Enhanced Intestinal IgM^+^ B Cell Count in Concert with a Strong Activation of the Intestinal Complement System

**DOI:** 10.3390/cells8010078

**Published:** 2019-01-21

**Authors:** Sophie Preisker, Ann-Kathrin Brethack, Arne Bokemeyer, Dominik Bettenworth, Christian Sina, Stefanie Derer

**Affiliations:** 1 Institute of Nutritional Medicine, Molecular Gastroenterology, University Hospital Schleswig-Holstein, Campus Lübeck, D-23538 Lübeck, Germany; sophie@preisker.de (S.P.); ann-kathrin.brethack@uksh.de (A.-K.B.); christian.sina@uksh.de (C.S.); 2 Department of Medicine B, Gastroenterology and Hepatology, University Hospital Münster, 48149 Münster, Germany; arne.bokemeyer@ukmuenster.de (A.B.); dominik.bettenworth@ukmuenster.de (D.B.)

**Keywords:** intestinal complement system, inflammatory bowel disease, Crohn’s disease, ulcerative colitis, complement receptor 2 (CR2), immunoglobulin M (IgM), B cells

## Abstract

Inflammatory bowel disease (IBD) is an umbrella term that comprises Crohn’s disease (CD) and ulcerative colitis (UC). Both entities are characterized by a disturbed mucosal immune response and an imbalance of intestinal microbiota composition. The complement system (C) plays a critical role in the detection, and clearance of bacteria and dysregulation of single complement components has been linked to IBD. Here, we asked if the C contributes to distinct subtypes of inflammation observed in CD and UC. We performed systematical expression analyses of the intestinal C in IBD patients and controls. Immunohistochemistry or immunoblot experiments were performed to verify qPCR data. Activity of the three activation pathways of C was studied in sera samples. In CD patients a strong upregulation of the C was observed enabling the definition of unique expression patterns being associated either with remission or active disease. These data were reflected by an enhanced C activation in sera and fecal samples. An excessive mucosal presence of immunoglobulin M (IgM) and CR2/CD21 positive B cells in concert with decreased fecal IgA level was identified in CD patients in remission. These findings point to an exacerbated induction of the intestinal C that may potentially be involved in the etiology of CD.

## 1. Introduction

Due to its main functions in detection, opsonization, and elimination of pathogens, as well as of apoptotic or malignant cells, the complement system is crucial for the efficient clearance of invading bacteria, as well as for intestinal tissue homeostasis [1]. However, uncontrolled and sustained complement activation evokes severe inflammatory processes and results in tissue damage, as seen in inflammatory bowel disease (IBD) [2]. Although IBD has been linked to genetic variants of genes belonging to the innate immune system, the exact etiology of IBD is still unresolved [3]. Ulcerative colitis (UC) is mainly restricted to the colon and presents severe mucosal inflammation that is accompanied by ulcerations. In contrast, Crohn’s disease (CD) is characterized by a discontinuous, transmural inflammation that may affect all layers of the intestine throughout the whole gastrointestinal tract [4]. Furthermore, there is growing evidence for diagnostic analysis of IBD-associated autoantibodies of the immunoglobulin gamma (IgG) or alpha (IgA) isotypes, e.g., against luminal antigens, such as glycoprotein 2 (GP2), to differentiate UC from CD patients, as well as distinct clinical phenotypes in CD [5]. While IgG mainly activates the classical pathway of the complement system via C1q binding, IgA has no C1q binding site but can activate the lectin, as well as the alternative pathway [6].

Interestingly, IgG-triggered classical complement activation has been detected on the intestinal epithelium of UC patients, while no C1q or C4c, but strong C3b deposition, was detected on the intestinal epithelium of CD patients [7]. Furthermore, higher C3 levels were detected in serum samples from CD patients in comparison to UC patients or healthy controls [8]. 

In chronic dextran sulphate sodium (DSS)-induced colitis, C1q^−/−^/ MBL^−/−^ mice died at the beginning of the experiment, while C5aR1^−/−^ or C3^−/−^ mice displayed stronger intestinal inflammation and decreased survival rates in comparison to wild type mice [9,10]. These findings further support the idea of the prominent role of the complement system in intestinal immune response during chronic inflammation.

Host cells typically express different complement regulatory proteins, such as CD46, CD55, or CD59, to protect themselves against uncontrolled deleterious effects of the complement system. While CD55 expression levels have been found to be strongly upregulated on the intestinal epithelial cells (IEC) of IBD patients with active disease, no changes could be detected for CD46 or CD59 [11]. Further studies confirmed these data and proposed analysis of CD55 expression levels in stool samples of UC patients as a useful marker of disease activity [12]. 

The available data suggest that mucosal complement activation exerts bactericidal activity, which is dysregulated in IBD. Furthermore, distinct interaction patterns between luminal bacteria and IECs, as well as intestinal immune cells in UC and CD patients, may be hypothesized as contributing to distinct subtypes of inflammation observed in CD and UC. Hence, the aim of the present study was to perform a systematical expression analysis of the intestinal complement system in IBD patients and control individuals.

## 2. Materials and Methods

### 2.1. Study Population

The study population of the present study included 119 individuals: 31 patients with histologically confirmed UC, 57 patients with CD, 10 control colitis patients (infectious and/or antibiotic-associated colitis), and 21 hospitalized normal (HN) without any evidence of intestinal inflammation. Patients characteristics are depicted in Table 1. Ileal and colonic biopsies, as well as the collection of serum or fecal samples, were obtained during or before colonoscopy, respectively, at the University Hospital Schleswig-Holstein, Campus Lübeck or the University Hospital Münster. Evaluation of acute flare of disease was based upon clinical data, endoscopic, and histological findings. In detail, the presence of active disease was considered either due to macroscopic and/or microscopic signs of intestinal inflammation. In the case that no endoscopy was available, active disease was considered on a paraclinical parameter, such as fecal occult blood, elevated CRP and leucocytes, and elevated fecal calprotectin levels, together with clinical signs of intestinal inflammation. This classification was carried out in consideration of the drugs used in the treatment of IBD, such as mesalamine, budesonide, prednisone, azathioprine, 6-mecaptopurine, and anti-TNFα antibody. The endoscopies were part of regular patient management. All patients agreed to participation by giving informed consent at least 24 h before the procedure, and the study was granted prior approval by the local ethics committees.

### 2.2. RNA Extraction and Real-Time Quantitative PCR

RNA was extracted using the innuPREP RNA Mini Kit (Analytik Jena AG, Jena, Germany) and transcribed to cDNA (RevertAid H Minus reverse transcriptase, Thermo Scientific, Schwerte, Germany) using the T Gradient thermocycler (Whatman Biometra, Göttingen, Germany). Real-time quantitative PCR (qPCR) was carried out using MaximaR SYBR Green qPCR Master Mix, plus specific oligonucleotides, using a 96-well plate format. The amplification program consisted of: (i) preincubation at 95 °C for 5 min; (ii) 40 cycles of denaturation at 95 °C for 45 s and annealing at appropriate temperature (55 °C) for 1 min, using the StepOne Plus Real-Time PCR System (Thermo Fisher Scientific, Darmstadt, Germany). Melting curve profiles were produced and analyzed following the ddCt algorithm. Expression level were normalized to β-Actin. The following oligonucleotides were used for analyses: β-Actin: for: 5′-ACATCCGCAAAGACCTGTACG-3′, rev: 5′-TTGCTGATCCACATCTGCTGG-3′; *C1QB*: for: 5′-ACCCCAGGGATAAAAGGAGAG-3′, rev: 5′-GGCAGAGAAGGC GATTTTCTG-3′.

In the case of the systematic expression analyses of 30 complement components/receptors/inhibitors by real-time PCR experiments, the following TaqMan arrays (Applied Biosystems, Foster City, CA, USA) were utilized: *C1QA* (Hs00381122_m1), *C1QB* (Hs00608019_m1), *C1QC* (Hs00757779_m1), *C1R* (Hs00357637_m1), *C1S* (Hs01043794_m1), *C2* (Hs00918862_m1), *C3* (Hs00163811_m1), *C4A* (Hs00416393_g1), *C5* (Hs00156197_m1), *C6* (Hs01110040_m1), *C7* (Hs00940408_m1), *C8A* (Hs00175098_m1), *C9* (Hs01036223_m1), *CFB* (Hs00156060_m1), *MBL2* (Hs00175093_m1), *MASP2* (Hs01548243_g1), *C3AR1* (Hs00377780_m1), *C5AR1* (Hs00383718_m1), *C5AR2* (Hs00218495_m1), *CR1* (Hs00559348_m1), *CR2* (Hs00153398_m1), *CR3* (Hs00355885_m1), *CR4* (Hs00174217_m1), *CD93* (Hs00362607_m1), *CALR* (Hs00189032_m1), *C1QBP* (Hs00241825_m1), *CD46* (Hs00611257_m1), *CD55* (Hs00892618_m1), *CD59* (Hs00174141_m1), *C4BP* (Hs00361221_m1), and *ACTB* (Hs99999903_m1). β-Actin served as the reference transcript. CT value from each transcript was normalized to actin beta (ACTB) value.

### 2.3. Immunohistochemistry

Immunohistochemical techniques were performed, according to standard protocols. Briefly, frozen tissue sections were fixed, cryostat sectioned and stained with a rabbit anti-human C1q antibody (A0136; Dako), a goat anti-human C3 antibody (sc-20137; Santa Cruz Biotechnology, Inc., Dallas, TX, USA), a rabbit anti-human CR2 (HPA052942, Sigma-Aldrich, St. Lous, MO, USA) or with respective isotype control antibodies, and then washed and incubated with HRP-conjugated anti-rabbit or anti-goat IgG secondary Abs. Afterwards, tissue slides were incubated with DAB substrate (Dako) and counterstained with Mayer`s hemalum solution.

### 2.4. SDS-PAGE and Immunoblotting

Whole-protein extracts were prepared by lysing biopsy or fecal samples in denaturing lysis buffer containing 1% SDS, 10 mM Tris (pH 7.4), and 1% protease inhibitor mixture (Complete Protease Inhibitor Cocktail; Roche Applied Science, Mannheim, Germany). Forty micrograms of protein extracts were separated by denaturing SDS-PAGE under reducing conditions and transferred onto polyvinylidene difluoride membranes. After blocking, the membranes were probed with C3-specific primary Ab (sc-20137, Santa Cruz Biotechnology, LLC, Solon, OH, USA) or a human IgM-specific primary Ab (A80-100A, Biomol, Hamburg, Germany), washed, and incubated with HRP-conjugated IgG as secondary Ab. The human IgA or IgG level was detected using HRP-conjugated IgG directed either against the human alpha chain (PA1-74395, Thermo Fisher Scientific) or against the human gamma chain (62-8420, Thermo Fisher Scientific). The proteins were visualized by chemiluminescence. To determine similar transfer and equal loading, the membranes were stripped and reprobed with an Ab specific for β-Actin (Sigma-Aldrich, St. Louis, MO, USA).

### 2.5. WIESLAB^®^ Complement Screen Assay

Human sera samples were collected from blood donors using the S-Monovette^®^ 1.6 ml Hirudin (Sarstedt, Nümbrecht, Germany). The activity of the classical, the alternative, and the lectin pathway of complement activation in human sera samples was determined utilizing the WIESLAB^®^ Complement Screen assay (Euro Diagnostica, Malmö, Sweden), according to the manufacturer’s instructions. 

### 2.6. Statistical Analysis

Data are displayed graphically and were statistically analyzed using GraphPad Prism 6.0. For the TaqMan array-based qPCR analyses, statistical significance was determined by the Fisher’s least significant difference (LSD) test. In the case of *C1QB* qPCR analysis, statistical significance was determined using the one-way test with the Holm-Sidak’s multiple comparison test. Statistical significance of data received from the WIESLAB^®^ Complement Screen assay or immunoblot experiments was determined by the Kolmogorov-Smirnov test. Values of *p* ≤ 0.05 were considered statistically significant. If not stated otherwise, experiments and mea­surements were replicated at least three times.

## 3. Results

### 3.1. Crohn’s Disease Patients in Remission Display an Upregulation of the Intestinal Complement System 

**Figure 1:**
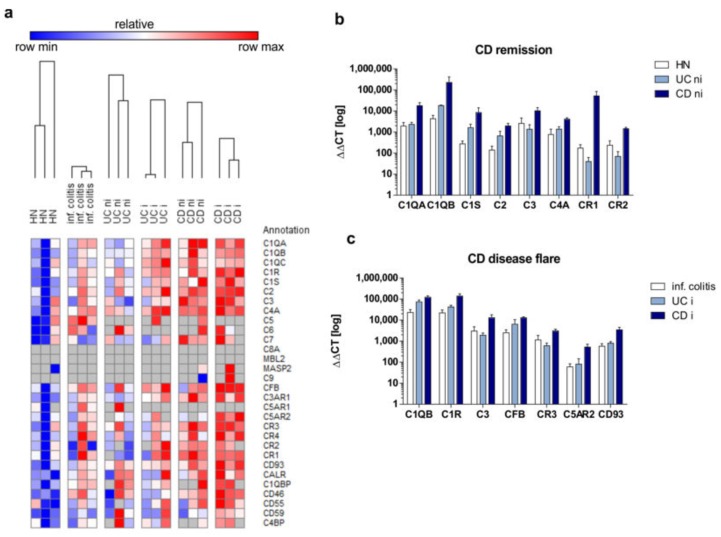
Complement components are more frequently expressed in Crohn’s disease (CD) patients than in ulcerative colitis (UC) patients. Real-time PCR analysis was performed utilizing customized TaqMan array plates for parallel amplification of 30 human complement components in non-inflamed in remission (ni) and inflamed (i) sigmoidal biopsy samples from CD and UC patients as well as from hospitalized normals (HN). Relative ddCt values are depicted in a heatmap (**a**) and subdivided into significantly regulated transcripts in remission state (**b**) or in active disease state (**c**). Results are expressed as means ± SEM.

To systematically study sigmoidal mRNA expression of the main 30 complement components, receptors or inhibitors in IBD patients or control individuals, we utilized target specific TaqMan arrays in real-time PCR experiments. As demonstrated in Figure 1a, mRNA expression of most complement system members could be amplified during qPCR experiments, while no mucosal mRNA expression of C8A, C9, MBL2, and MASP2 was detected in any of the tested cDNA samples (Figure 1a). In sigmoidal cDNA samples from HN, mRNA expression level of analyzed transcripts were ranked in the following order: *CALR*, *C1R*, *C1QB*, *CD55*, *C3*, *CD59*, *C1QA*, *C1QC*, *C4A*, *CD46*, *CR4*, *CR3*, *CFB*, *C1S*, *C7*, *CR2*, *CR1*, *CD93*, *C2*, *C4BPB*, *C3AR*, *C5AR2*, *C6*, *C1QBP*, *C5*, and *C5AR1*. Notably, the expression level of the following mRNA transcripts was significantly upregulated in CD patients in remission when compared to HN: *C1QA* (9-fold), *C1QB* (53-fold), *C1S* (31-fold), *C2* (14-fold), *C3* (3-fold), *C4A* (5-fold), *CR1* (304-fold), and *CR2* (6-fold) (Figure 1b). The highest mucosal mRNA expression in CD patients in remission was detected for *C1QB*, *CR1*, and *C1QA,* followed by *C1S*, *C2*, *C3*, *C4A,* and *CR2*, respectively. Furthermore, under active disease conditions, the expression level of following mRNA transcripts was significantly upregulated in inflamed sigmoidal biopsy samples from CD patients in comparison to colitis control patients: *C1QB* (5-fold), *C1R* (6-fold), *C3* (4-fold), *CFB* (5-fold), *CR3* (3-fold), *C5aR2* (8-fold), and *CD93* (6-fold) (Figure 1c). None of the tested transcripts was significantly altered in UC patients in remission nor in UC patients with active disease. 

**Figure 2:**
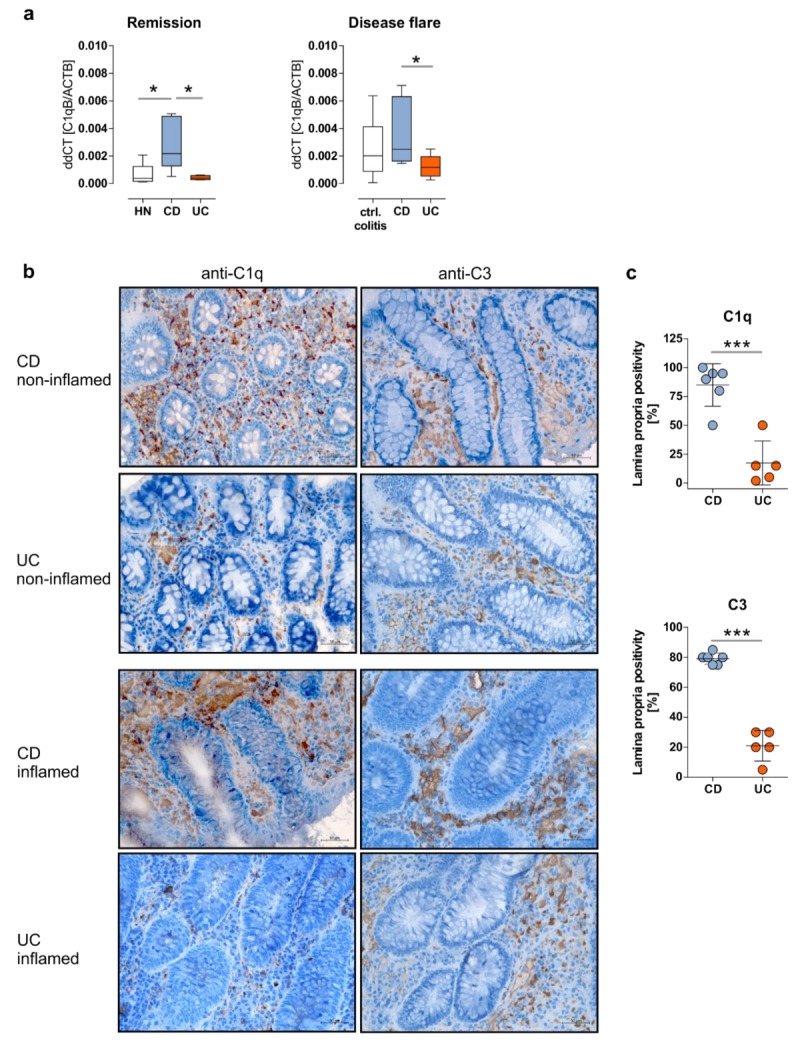
CD patients in remission display an over-representation of mucosal C1q and C3 expression. (**a**) The mRNA expression of *C1QB* in non-inflamed (ni) sigmoidal biopsy samples from IBD patients in remission or HNs, as well as from inflamed sigmoidal biopsy samples from IBD patient with active disease or control colitis patients was analyzed by qPCR using *C1QB* specific oligonucleotides. ddCt values are depicted. * *p* ≤ 0.05. (**b**) Representative pictures of C1q (left panels) or C3 staining (right panels) in colonic tissues from CD or UC patients (non-inflamed or inflamed) (original magnification: 20×). (**c**) Percentages of C1q or C3 positivity of the lamina propria were determined in analyzed CD (n = 6) or UC (n = 5) tissue samples, irrespectively of the inflammatory status. *** *p* ≤ 0.001.

In the next step, we validated results received from real-time PCR experiments by qPCR experiments utilizing target-specific primer pairs in combination with different sigmoidal cDNA samples, as well as by immunohistochemistry analyses. As presented in Figure 2a, we corroborated enhanced CD-specific expression of *C1QB* in remission and active disease by qPCR analyses. In remission, *C1QB* mRNA expression was significantly upregulated in cDNA samples from CD patients (n = 7) in comparison to UC patients (n = 4), as well as to HN (n = 5)’ however, while in active disease, *C1QB* mRNA expression was only enhanced in CD patients in comparison to UC patients (Figure 2a). Recently, a sigmoidal *C3* upregulation during acute flare and remission was found in CD patients, but not in patients with UC nor in disease controls [13]. Hence, these data are not included in the present manuscript.

In line with these findings, immunohistochemistry (IHC) analyses indicated a strong expression of C1q and C3 proteins in the lamina propria of CD, while very low C1q or C3 protein levels were present in UC patients. Interestingly, in CD patients, no obvious differences in C1q or C3 staining intensities were seen between non-inflamed (remission) and inflamed (active disease) biopsy samples (Figure 2b). Quantification of C1q or C3 positivity of the lamina propria revealed a statistically significant higher C1q and C3 expression in CD patients when compared to UC patients, irrespective of the inflammatory status of the analyzed biopsy samples (Figure 2c). 

### 3.2. Enhanced Activation of the Complement System Can be Monitored in Serum and Feces Samples in CD Patients

**Figure 3:**
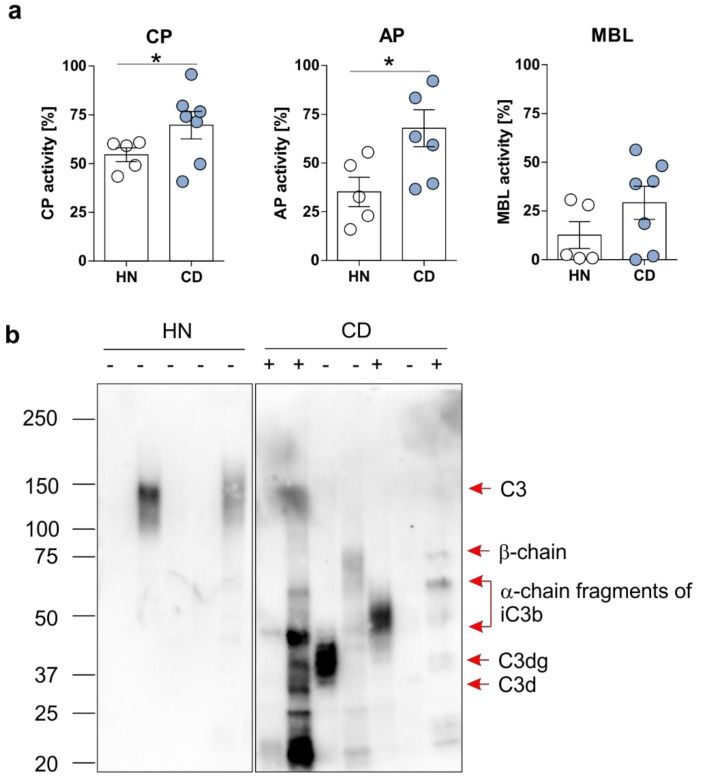
Activity of the classical and the alternative pathways of complement activation is enhanced in CD patients in remission. (**a**) The activity of the three complement activation pathways (CP, AP, and MBL) was studied in sera samples collected from hospital normal (HN; n = 5) or CD patients in remission (n = 7) using the WIESLAB® Complement Screen assay. Mean ± SEM values are depicted. * *p* ≤ 0.05. (**b**) C3 protein expression and cleavage was analyzed in fecal samples collected from HN (n = 5) or CD patients (n = 7) by immunoblot experiment using a primary antibody specific for C3 and its cleavage fragments C3b, C3d, C3g. + = disease flare, − = remission.

Based on findings received from mRNA, as well as protein expression analyses described above, the activity of the three different pathways of complement activation was investigated in serum samples from CD patients in remission and HNs. As expected, CD patients in remission displayed a significantly enhanced activity of the classical (CP; 1.3-fold), as well as the alternative (AP; 2-fold), pathways of complement activation in serum in comparison to HN (Figure 3a). However, activity of the MBL was not significantly altered. 

Recently, our group published results demonstrating a correlation between fecal C3 level and disease activity, as well as the opsonization of mucosa-associated bacteria with C3 cleavage fragments, in a mouse model of experimental chronic colitis [13]. Hence, we tested the presence of cleavage fragments of C3 in feces samples collected from CD patients in remission, as well as from HN in the present study. As depicted in Figure 3b, low protein levels of full-length C3 were detected by western blot experiment in fecal samples from HN. However, high protein level of C3 cleavage fragments were found in fecal samples from CD patients that did not correlate with disease activity (Figure 3b). Notably, the highest C3 (b/d/g) level was observed in fecal samples collected from CD patients with an ileal manifestation of disease (patient #2, #3, and #5). While CD patients #2 and #3 were reported to display exclusively an ileitis, CD patient #5 has been reported to display an ileitis in concert with a pancolitis. In the case of all other analyzed CD patients, an ileocolitis (#1, #6, and #7), as well as an isolated colitis (#4), was documented.

### 3.3. The Mucosal B Cell Compartment is Overrepresented Exclusively in CD Patients in Remission 

**Figure 4:**
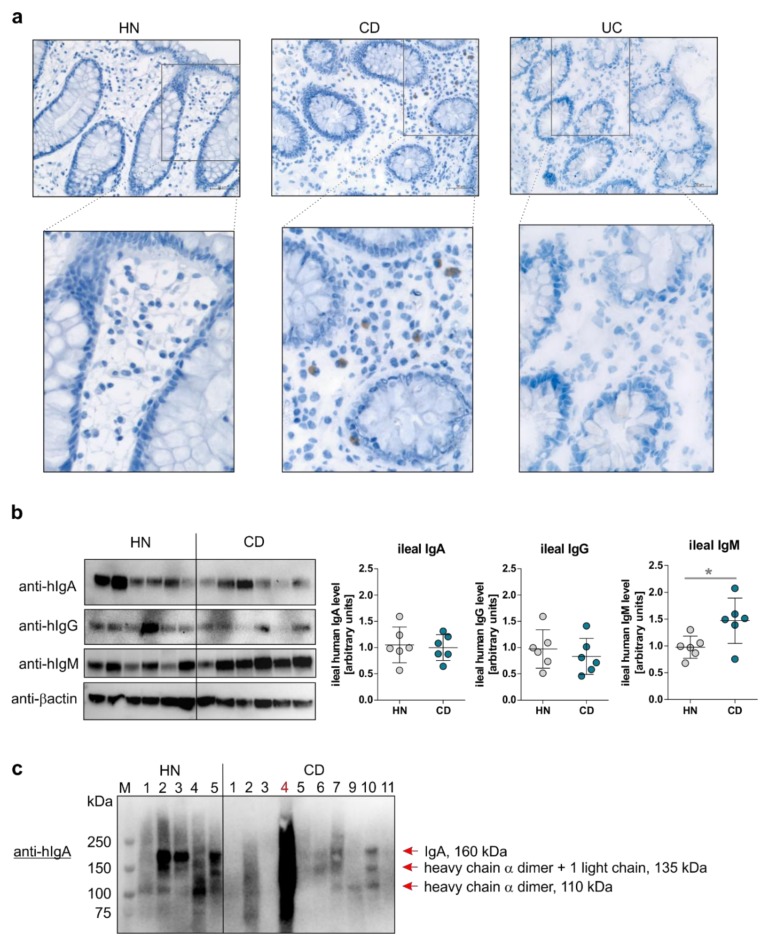
Intestinal naïve memory B cells are highly present in CD patients in remission. (**a**) CR2 protein level in colonic biopsy samples from HN or CD, as well as UC patients in remission, was determined by immunohistochemistry (IHC) analysis. Representative pictures are presented (original magnification: 20×). (**b**) Western blot analyses of immunoglobulin alpha (IgA), human immunoglobulin gamma (IgG), or immunoglobulin M (IgM) expression in ileal biopsy samples from HN (n = 6) or CD patients in remission (n = 6). Western blot analysis of β-Actin served as a loading control (left panel). Densitometric analysis of whole hIgA, hIgG, or hIgM protein expression utilizing the software ImageJ (right panel). Mean ± SEM values are depicted. * *p* ≤ 0.05. (**c**) Western blot analysis of hIgA expression in fecal samples from HN (n = 5) or CD patients in remission (n = 11; CD patient #4 is in active disease).

Besides an enhanced mucosal mRNA expression of *C1QA*, *C1QB*, *C1S*, *C2*, *C3*, and *C4A*, mRNA transcripts of complement receptors *CR1* and *CR2* were also identified in the present study to be exclusively upregulated in the mucosa of CD patients in remission (Figure 1b). Due to a B-cell and/or follicular dendritic cell specific expression pattern of CR2, in concert with unaffected *CR3* and/or *CR4* mRNA expression, highly expressed on macrophages and granulocytes, an overrepresentation of the B-cell compartment in CD patients was hypothesized. Hence, we performed immunohistochemical staining experiments of mucosal CR2 positive cells in the lamina propria (LP) of HN and IBD patients in remission. As expected from qPCR analyses, a higher proportion of CR2 positive immune cells was observed in the LP of CD patients in comparison to UC patients, as well as of HN (Figure 4a). In addition, to study functional consequences of high mucosal CR2 positive B-cell count detected in CD patients, we analyzed ileal and fecal human immunoglobulin level in HN, as well as in CD patients, by immunoblot experiments. Interestingly, no differences were detected in ileal IgA nor IgG level between CD patients and HN, while ileal IgM levels were significantly enhanced in CD patients (Figure 4b). Most strikingly, CD patients in remission displayed lower fecal IgA levels in comparison to HN, pointing, together with the observation of increased IgM positive B-cell counts, to a disturbed induction of IgA secreting plasma cells in CD patients (Figure 4c). 

## 4. Discussion

In the present study, an exacerbated activation of the intestinal complement system that does not significantly differ between remission and active disease state was observed in CD but not in UC patients. 

Although it still remains to be elusive which intestinal cell entity drives excessive intestinal complement system expression in CD, highly complement expressing macrophages can be excluded due to no increased expression level of CR3 and CR4 in CD patients in remission. Based on data indicating enhanced prevalence of adhesive-invasive *E. coli* in the intestine of CD patients [14] combined with recently published data by our group, high intestinal C3 expression in CD may be hypothesized to be a result of over-activation of the TLR4 signaling cascade in IECs under quiescent disease state [13]. Complement and Toll-like receptors (TLRs) are two cross-talking sensor and effector systems critical for bacterial defense. While both systems independently recognize microbial-associated molecular patterns (MAMPs), they may synergize to amplify and regulate antimicrobial and inflammatory immune response [1]. For example, C3aR signaling enhances LPS-induced IL-1β secretion via inflammasome activation in monocytes [15]. These results were further reinforced by data from our group, demonstrating that the IECs constitutively produced C3 and expressed C3aR, as well as TLRs1-4. Importantly, C3 expression patterns differed between ileum and colon, with significantly higher C3 expression levels in the colon with this spatial difference being induced or maintained through commensal bacteria. In addition, TLR4-mediated signals in IECs induced increased C3 expression and C3a generation, which in turn led to an enhanced ratio of pro- to anti-inflammatory cytokines. We confirmed these findings in primary IEC from chronic DSS-induced colitis models. High amounts of C3 were also detected in murine mucus samples, leading to opsonization of bacteria with C3 fragments to ensure proper presentation to antigen-presenting cells, such as macrophages, dendritic cells, or B cells [13]. 

In line with these findings, the analyses of mucosal complement receptor expression patterns in the present study revealed the B cell compartment, characterized by CR2 (also CD21) and CR1 (also CD35) expression [16], to be over-represented in CD patients. Of note, we identified in CD patients an expansion of mucosal IgM^+^/CR2^+^ B cells, while IgA secreting plasma cells seemed to be under-represented, as indicated by decreased fecal IgA level.

Notably, Sieber et al. published in 1984 that lymphocytes, when isolated from CD patients in comparison to healthy individuals, display a higher spontaneous secretion of immunoglobulins, while the induction of immunoglobulin secreting plasma cells by pokeweed mitogen stimulation of these lymphocytes was impaired [17]. Other studies also reported a hyperactivation of B cells in CD but not UC patients, mainly characterized by highly phosphorylated Bruton’s tyrosine kinase (BTK) [18] or by over-expression of lymphocyte activation antigens, such as the transferrin receptor or IL-2 receptor [19]. Notably, a recent study demonstrated B cells to be more present than immunoglobulin secreting plasma cells in the intestine of CD patients. Additionally, circulating CD21^low^CD38^−^ B cells (mainly IgM positive) were increased and CD27^+^IgM^+^IgD^+^ natural effector B cells were reduced, while the number of class-switched B cell subtypes and plasma blasts was not altered [20]. 

**Figure 5:**
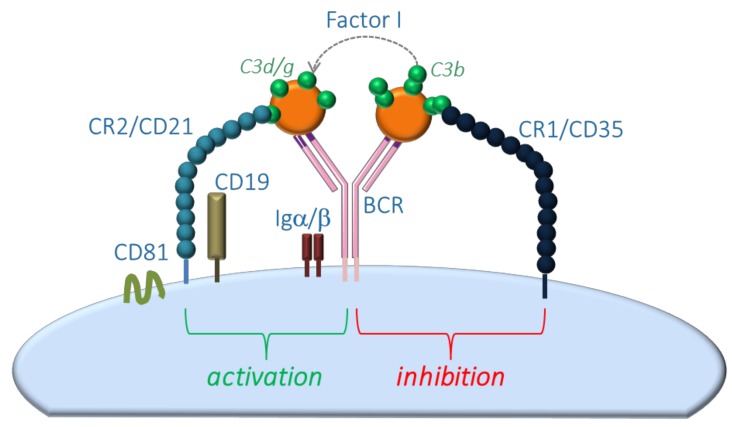
Model scheme of complement-mediated activation of mucosal naïve/memory B cells.

The role of CR1 and CR2 ligation by C3 cleavage fragments C3b/d/g in B cell activation was demonstrated in in vivo mouse models using mice lacking CR1/CR2 expression on B cells but not on follicular dendritic cells. These mice displayed a defect in the induction of T-cell dependent antibody responses [21]. Further functional studies identified CR2 to be a member of the co-receptor complex of the B-cell receptor complex together with CD19 and CD81 [22]. Binding of this co-receptor complex to C3d on coated antigens by CR2 in concert with antigen binding by the B-cell receptor lowered the threshold for B-cell activation up to 1,000-fold, leading to reduced antigen requirement for proper B-cell activation. Besides CR2, CR1 was also expressed on B cells and bound the opsonin C3b. Interestingly, crosslinking of CR1 on B cells by C3b-opsonized antigens inhibited B-cell proliferation [23,24]. However, in the presence of factor I, CR1 was able to cleave C3b to generate C3d and, therefore, provide the ligand for CR2-mediated B-cell activation (Figure 5). 

However, under conditions where early and late complement components were present, the formation of the membrane-attack complex (MAC) and hence B-cell lysis occurred. While CR2 blockade by specific antibodies prevented C3-fragment deposition on B cells, antibody-mediated blockade of CR1 resulted in reduced C3-fragment deposition and reduced MAC formation, pointing to a significant role of CR1 in triggering activation of the whole complement cascade on the cell surface of B cells [25]. Of note, mice lacking CR1/2 display impaired humoral responses to T-cell dependent antigens that were reflected by reduced antibody titers in serum and reduced numbers of germinal centers [26,27]. These data underline the hypothesis that the complement system, as well as the complement receptors CR1 and CR2, is crucial for the induction of a proper humoral immune response. 

Besides high mucosal CR1, CR2, and C3 expression in CD patients, the initial complement component of the classical pathway of complement activation C1q was identified in the present study to be strongly upregulated on mRNA and protein level in CD patients already in remission. This is in contrast to data published by Halstensen et al. demonstrating no C1q IHC staining in the intestine of CD patients [7]. One explanation for this discrepancy may be the different staining protocol utilized in immunohistochemistry experiments by Halstensen et al. For example, they only stained intestinal C1q protein in the presence of anti-C3b or anti-IgG antibodies, while in the present study single staining protocols were utilized. Furthermore, in the study from Halstensen et al., an anti-C1q rabbit serum was utilized versus a polyclonal rabbit IgG directed against human C1q utilized in the present study. Together, distinct staining techniques combined with different primary anti-C1q antibodies may result in distinct results. While C1q is highly expressed by macrophages, it has been demonstrated to mainly bind to C1q receptors expressed on the cell surface of monocytes/macrophages, B cells, and polymorphonuclear cells, as well as to different immunoglobulin isotypes, such as IgG and IgM [6,28]. Interestingly, C1q binding to activated B cells has then been unraveled to enhance secretion of immunoglobulins like IgA, IgG, or IgM [29,30]. Furthermore, especially IgM expressing B cells were identified to trigger C1q-dependent complement activation on their cell surfaces under conditions that enable previous crosslinking of the IgM-B cell receptor [31,32]. In line with these findings, Rossbacher and Shlomchik reported loss of C3 fragment deposition on B cells, as well as impaired formation of functional germinal centers, in mice expressing mutant IgM on the cell surface of B cells that lacks C1q binding capacity [33]. 

Together, CD patients in remission displayed increased intestinal IgM expressing B cells in concert with early classical complement pathway components. Hence, it may be hypothesized that excessive intestinal C1q and C3 level trigger crosslinking of the IgM-B-cell receptor via C1q binding, as well as proper B-cell activation via C3 binding to CR1 and CR2, resulting in an exacerbated induction of the humoral immune response in CD patients. Further investigations shall be performed to link these observations to the presence of high autoantibody titers that occur mainly in CD but not in UC patients. Additionally, data presented in the current study reveal the analysis of fecal C3 level as a potentially novel screening method to further discriminate between CD and UC or between CD and non-IBD colitis, independently of status or localization of the disease.

## Figures and Tables

**Table 1:** Overview of study population.

		Male [n]	Female [n]	Disease Localization[n]	Biopsy Localization [n]	Medication [n]
**qPCR** **TaqMan arrays** [n = 18]	*HN* *ctrl.Colitis*	*2* *2*	*1* *1*	n.a. PC [2], PrC [1]	S [2], cd [1] S [3]	n.a. P [1], M [1]
	*CD_ni*	*0*	*3*	PC [2], IC [1]	S [3]	P [2], Su [1], A [2], MD [1], CB [1]
	*CD_i*	*2*	*1*	PC [1], PrC [2]	S [3]	P [2], M [2], A [2], T [1]
	*UC_ni*	*1*	*2*	C [2], PrC [1]	S [3]	M [2], A [1]
	*UC_i*	*0*	*3*	PC [1], PrC [2]	S [3]	P [3], M [1], A [1], MD [2]
**qPCR** ***C1qB*** [n = 38]	*HN* *Ctrl. colitis* *CD_ni*	*2* *2* *2*	*3* *5* *5*	n.a. C [7] IC [4], C [2], PC [1]	ct [2], cd [2], S [1] ce [1], ct [2], S [3], R [1] ca [2], ct [1], S [4]	CB [1] M [2], Su [1], MD [2], CB [2] P [3], B [1], M [4], Su [1], A [3], CP [1], MD [2], CB [1]
	*CD_i*	*1*	*4*	IC [2], C [2], PC [1]	ce [1], ca [2], cd [1], S [1]	P [1], B [1], M [2], Su [1], A [3], Am [1], MD [2]
	*UC_ni*	*3*	*1*	C [2], PrC [2]	S [4]	P [1], M [2], A [3]
	*UC_i*	*5*	*5*	C [3], PC [6], PrC [1]	cd [2], S [7], R [1]	P [2], M [7], Su [1], A [4], MP [1]
**SDS-** **PAGE** [n = 25]	*HN* *CD_ni* *CD_i*	*2* *3* *2*	*4* *12* *2*	n.a. I [2], IC [8], C [5] I [2], IC [2], PC [1]	ti [6], fe [5] ti [6], fe [9] fe [4]	n.a. P [3], B [1], M [7], Su [1], A [4], T [11], MD [1] B [1], M [1], T [1]
**IHC** [n = 25]	*HN* *CD_ni* *CD_i* *UC_ni* *UC_i*	*n.a.* *4* *2* *3* *1*	*n.a.* *6* *1* *4* *3*	n.a. I [1], IC [2], C [5], PC [2] C [1], PrC [1], PC [1] C [3], PrC [3], PC [1] C [1], PrC [1], PC [2]	S [1] ca [3], ct [2], S [4], R [1] ca [1], S [2] ti [1], ce [1], ca [1], cd [2], S [2] cd [2], S [2]	n.a. P [2], B [1], M [3], Su [2], A [3], MD [1], CB [1] P [1], M [2], Su [1], A [1] P [3], M [6], Su [1], A [4] P [2], M [3], Su [1], TL [1], MD [1], CB [1]
**Complement** **ELISA** [n = 13]	*HN* *CD_ni*	*3* *5*	*3* *2*	n.a. I [3], IC [3], C [1]	NHS [6] NHS [7]	n.a. P [1], B [1], M [4], A [2], T [7]

HN = hospitalized normals, ctrl. Colitis = control colitis, CD = Crohn’s disease patients, UC = ulcerative colitis patients, i = patients with inflammation and active disease, ni = patients with no inflammation and in remission, n = number of patients, n.a. = not applicable, ti = terminal ileum, ce = cecum, S = sigma, cd = colon descendens, ca = colon ascendens, Ct = colon transversum, R = rectum, fe = feces, PC = pancolitis, PrC = proctocolitis, I = ileitis, IC = ileocolitis, C = colitis, P = Prednisone, B = Budesonide, M = Mesalamine, Su = Sulfasalazine, A = Azathioprine, CP = Cyclophosphamide, TL = Tacrolimus, MD = Metronidazole, CB = Ciprobay, T = anti-TNFα antibody, Am = Amox, MP = 6-Mercaptopurine, and NHS = normal human serum.
